# High intensity focused ultrasound inhibits melanoma cell migration and metastasis through attenuating microRNA-21-mediated PTEN suppression

**DOI:** 10.18632/oncotarget.10433

**Published:** 2016-07-06

**Authors:** Huan Li, Shi-mei Yuan, Min Yang, He Zha, Xue-ru Li, Hui Sun, Liang Duan, Yue Gu, Ai-fang Li, Ya-guang Weng, Jin-yong Luo, Tong-chuan He, Yan Wang, Chong-yan Li, Fa-qi Li, Zhi-biao Wang, Lan Zhou

**Affiliations:** ^1^ Key Laboratory of Clinical Diagnosis of Education Ministry, College of Laboratory Medicine, Chongqing Medical University, Chongqing 400016, China; ^2^ State Key Laboratory of Ultrasound Engineering in Medicine Co-founded by Chongqing and The Ministry of Science and Technology, Chongqing Key Laboratory of Ultrasound in Medicine and Engineering, College of Biomedical Engineering, Chongqing Medical University, Chongqing 400016, China; ^3^ Molecular Oncology Laboratory, Department of Orthopaedic Surgery and Rehabilitation Medicine, The University of Chicago Medical Center, Chicago, IL 60637, USA

**Keywords:** High intensity focused ultrasound (HIFU), melanoma, metastasis, microRNA-21, PTEN

## Abstract

High intensity focused ultrasound (HIFU) technology is becoming a potential noninvasive treatment for solid tumor. To explore whether HIFU can be applied to treat melanoma and its metastasis, we investigated the effect of HIFU on murine melanoma model. While there was little influence on cell survival, viability or apoptosis, HIFU exposure suppressed melanoma cell migration *in vitro* and metastasis *in vivo*. The expression of microRNA-21(miR-21) was down-regulated and PTEN expression was up-regulated in response to HIFU exposure, which was in concomitant with the reduction of AKT activity. Furthermore, ectopic miR-21 expression suppressed this effect of HIFU. These results demonstrate that HIFU exposure can inhibit AKT-mediated melanoma metastasis via miR-21 inhibition to restore PTEN expression. Therefore, targeting the miR-21/PTEN/AKT pathway might be a novel strategy of HIFU in treatment of melanoma.

## INTRODUCTION

Melanoma is an extremely aggressive skin cancer due to its high metastatic potential. Although it is a relatively uncommon cancer in China, it accounts for over 65% of skin cancer-related deaths [1]. Surgery is effective in carefully selected patients with primary tumor, but nonsurgical approaches have low response rates among patients at late stages [2, 3]. Metastasis is a major cause of treatment failure and mortality for melanoma. Thus, new therapeutic strategies to suppress melanoma metastasis are needed [4].

High intensity focused ultrasound (HIFU) technology is used in focused ultrasound surgery (FUS). Due to its noninvasiveness and lower complication risk, it is increasingly applied to treat solid tumors including breast cancer, hepatoma, pancreatic cancer, bone tumor and nephroma [5, 6]. HIFU exposure can effectively kill tumor cells by leading to thermal coagulative necrosis, and physical alteration such as cavitation, mechanical effect. It can alleviate the tumor burden of the host and boost the host immunity [7]. However, the effect of HIFU exposure on distant metastasis has not been well investigated.

MicroRNAs (miRNAs) are short (20-22 nucleotides) evolutionarily conserved endogenous non-coding ribonucleotidic acids that partially bind to complementary recognition sequences of mRNA to post-transcriptionally regulate gene expression [8, 9]. It was found that miRNAs play a role in the pathogenesis of various human disorders including skin diseases and that targeting specific pathways by miRNAs has therapeutic potential in diverse pathologies [10, 11].

A previous study from our research has demonstrated that HIFU can enhance host anti-tumor immunity by inhibiting the negative regulatory effect of microRNA-134 on CD86 in a murine melanoma model. And it has been reported that microRNA-21 have important roles in noninvasive physical therapy such as radiation recently [12, 13]. While HIFU treatment has emerged as a new physical therapeutic modality, whether miRNA-21 plays a role in the anti-cancer effect of HIFU has not been determined.

In this study, we investigated the effect and mechanism of HIFU in suppressing murine melanoma B16-F10 cell migration and metastasis. The results show that HIFU exposure effectively inhibited migration *in vitro* and metastasis *in vivo*, which was associated with suppression of microRNA-21, increase of its target gene phosphatase and tensin homolog deleted on chromosome ten (PTEN), and inactivation of AKT in the melanoma cells. Our study identifies a miR-21/PTEN/AKT pathway involved by HIFU, which may be implicated in treatment of melanoma.

## RESULTS

### HIFU exposure inhibited migration in B16-F10 cells

**Figure 1:**
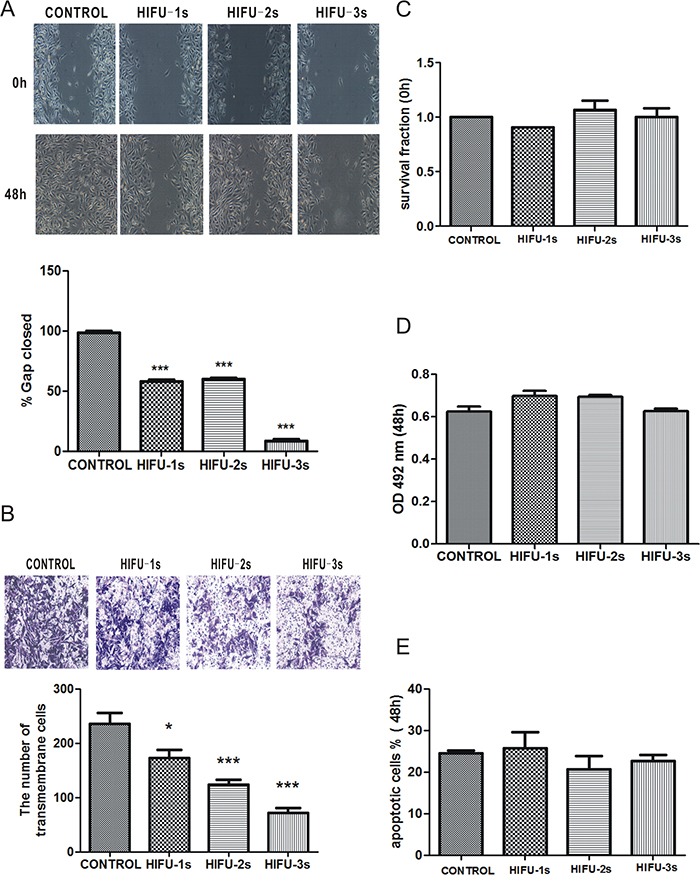
HIFU exposure inhibited migration in B16-F10 cells **A.** Wound healing assay for analyzing the effect of HIFU on migration of B16-F10 cells. The incision width of different sites was measured, and average healing rate was calculated. ***p<0.001. The representative images are shown in the upper panel, and average healing rate was showed in the bottom panel. **B.** Cell migration was detected by Transwell migration assay. Magnification, ×100. The representative images are shown in the upper panel, and three independent experiments are quantified in the lower panel. *p<0.05, ***p<0.001. **C.** Cells survival determined by Trypan blue exclusion assay. **D.** Cell viability was measured using the MTT assay. **E.** The percentage of cells undergoing apoptosis measured by flow cytometry analysis.

Firstly we investigated the effect of HIFU on migration of B16-F10 cells *in vitro* by wound healing assay with treating the cells with HIFU for 1 to 3 seconds. The results showed that the gap closure rates of control, HIFU 1s, HIFU 2s, and HIFU 3s groups at 48h were 98.33%, 57.94%, 59.91%, and 8.67 %, respectively (p<0.001. Figure 1A). The inhibitory effect on cell migration by HIFU exposure was further confirmed by transwell migration assay, which showed that the migrated cell number in the HIFU groups was increased by 26.77%, 47.51%, 69.41% compared to that of the control group (p<0.05, p<0.001, p<0.001. Figure 1B). However HIFU exposure had little impact on cell viability and apoptosis (Figure 1C, 1D, 1E).

### HIFU exposure suppressed miR-21 expression and increased PTEN in B16-F10 cells

**Figure 2:**
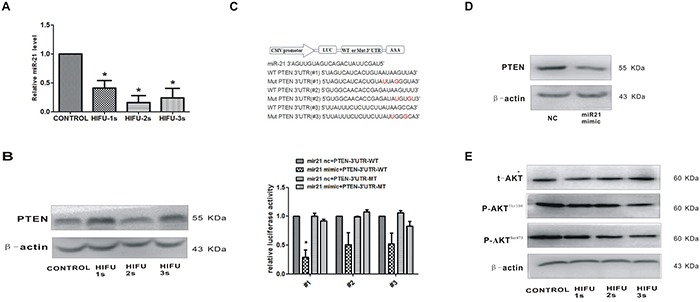
HIFU exposure decreased miR-21 expression and increased its target gene PTEN in B16-F10 cells **A.** Detection of miR-21 relative expression in B16-F10 cells after HIFU exposure by qPCR.*p<0.05. **B.** The expression of PTEN in B16-F10 cells after HIFU exposure were tested by Western blot. β-actin was used as an input control. **C.** PTEN was confirmed as a miR-21 target in B16-F10 using luciferase reporter assay. The cDNA sequence are shown in the upper panel, and the results of luciferase reporter assay were showed in the bottom panel. *p<0.05. **D.** The reduction of PTEN protein levels by transfecting with miR-21 mimic using Western blot. **E.** The expression of AKT and p-AKT after HIFU exposure was detected by Western blot. β-actin was used as a internal reference control.

Recent studies suggest that down-regulation of miR-21 could function as an effective approach for increasing radiosensitivity in cancer cells [18–20]. MiR-21 is an important oncogenic miRNA that is closely related to melanoma metastasis [21–23]. We found that miR-21 expression in B16-F10 cells was decreased after HIFU treatment (p<0.01, Figure 2A). Meanwhile, PTEN, the potential downstream gene of miR-21, was up-regulated (Figure 2B).

### miR-21 directly targeted PTEN in B16-F10 melanoma cell

Next, we investigated whether PTEN was a direct target gene of miR-21 in B16-F10 cells by using a firefly luciferase reporter assay. The three regions of the PTEN-3′-UTR mRNA were cloned into the pMIR-REPORT luciferase vector downstream of the firefly luciferase gene. A statistically significant decrease of luciferase activity was observed in the B16-F10 cells co-transfected with miR-21 mimic and pMIR-PTEN (p<0.05), compared with that of negative control. Importantly, miR-21 did not impact the luciferase activity of pMIR-PTEN/mut that has lost the miR-21-binding site (Figure 2C). The converse correlation of miR-21 to PTEN protein expression was confirmed by Western blot analysis in B16-F10 cells transfected with miR-21 mimic (p<0.05, Figure 2D). Taken together, these results suggested that PTEN-3′-UTR has the direct binding site of miR-21, which functions in miR-21-mediated PTEN up-regulation in B16-F10 cells after HIFU exposure.

The AKT kinase is the main downstream effector of PTEN. Accompanied with the increased expression of PTEN after exposure to ablative HIFU, the expression of the active form of the AKT, pAKT (Thr308) and pAKT (Ser473), was decreased (Figure 2E). These results confirmed that HIFU suppresses the AKT pathway by the increased PTEN expression.

### miR-21 suppression inhibited the migration of B16-F10 melanoma cells through up-regulation of PTEN after HIFU exposure

**Figure 3:**
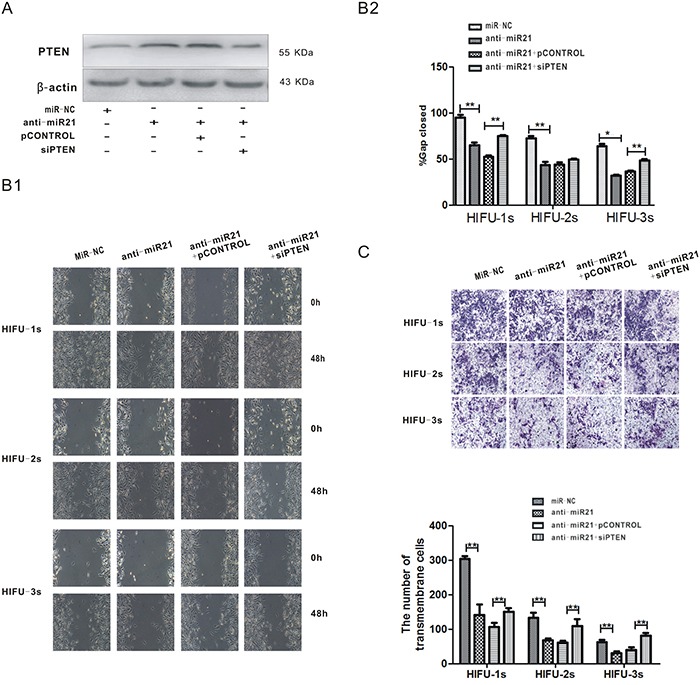
The decrease of miR-21 inhibited the migration of B16-F10 melanoma cells through up-regulation of PTEN after HIFU exposure **A.** B16-F10 cells were co-transfected with anti-miR-21 and psiPTEN plasmid, PTEN protein level was measured by Western blot analysis after 48 h. **B.** After transfection, the migration of residual B16-F10 cells was detected by wound healing assay. The representative images are shown in the left bottom panel (Figure B1), and quantification of cell migration was done by averaging the gap distances then showed in the right upper panel (Figure B2). **C.** Transwell migration assay was used to detect cell migration after the transfection. Magnification, ×100. The representative images are shown in the upper panel, and the numbers of transmembrane cells per microscopic field of three independent experiments are quantified in the bottom panel.**p<0.001.

To investigate the relationship between miR-21 down-regulation and HIFU-induced anti-metastatic effects, we knocked down miR-21 expression by introducing miR-21 inhibitor into B16-F10 cells (Figure 3A) and tested the change of cell migration activity by wound healing and transwell assays after HIFU exposure. After transfection of miR-21 inhibitor for 48 h, the wound closure rates of the three HIFU exposed groups were decreased by 31.6%, 39.9% and 50.4%, and the cell number of transmembrane migration was significantly decreased by 53.1%, 43.3%, 44.7%, all compared with those of the negative control group (p<0.01, Figure 3B, 3C). These data suggested that the down-regulation of miR-21 was involved in HIFU-induced anti-migration effect.

Then we performed a reversal experiment by co-transfecting the B16-F10 cells with miR-21 inhibitor and psiPTEN, before HIFU exposure. The miR-21 inhibitor increased PTEN protein expression in B16-F10 cells (Figure 3A), which was associated with migration inhibition (Figure 3B, 3C). A converse effect was seen in psiPTEN–transfected cells (Figure 3B, 3C). These results suggest that miR-21 inhibits the migration of B16-F10 cells via up-regulating PTEN, which was induced by HIFU exposure.

### HIFU exposure suppressed melanoma metastasis, which was associated with suppression of miR-21 expression in a murine melanoma model

**Figure 4:**
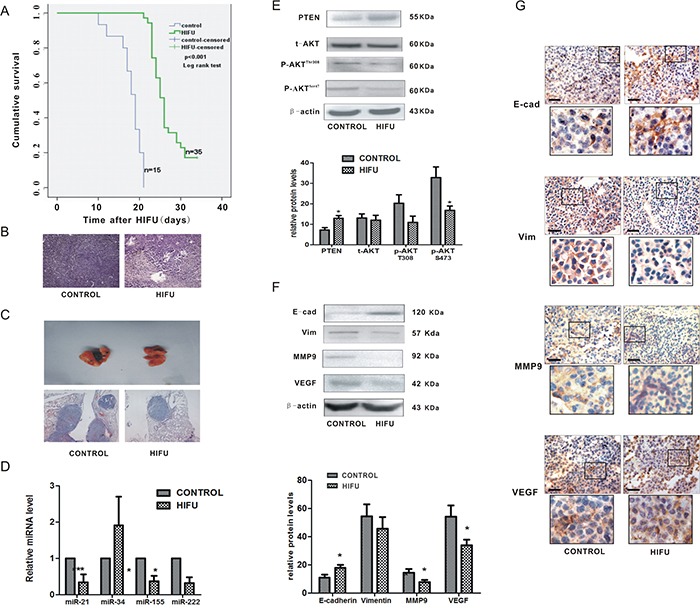
HIFU exposure suppressed melanoma metastasis and down-regulated miR-21 expression *in vivo* **A.** The cumulative survival curves in both groups using Kaplan-Meier analysis. **B.** Representative images of histological tissue sections of mice 14 days after HIFU or sham-HIFU exposure. Magnification, ×100. **C.** Representative H-E staining images of lungs of mice after subcutaneous inoculation of B16-F10 melanoma cells and treated with HIFU or sham-HIFU are shown in the upper panel. ×100. **D.** The alteration of microRNAs relative expression was detected by qPCR in residual tumor tissues after HIFU exposure. ***p<0.001, *p<0.05. **E.** The expression of PTEN, AKT and p-AKT in the residual tissues after HIFU exposure were detected using Western blot analysis in the upper panel. β-actin was used as an internal reference control. The quantified relative expression of proteins were showed in the lower panel.*p<0.05. **F.** The proteins in residual tumor tissues were detected by Western blot. β-actin was detected as an input control. The densitometric ratios are shown in the lower panel.*p<0.05. **G.** Immunohistochemical analysis was used to investigate the relative expression of proteins involved in metastasis. Scale bar, 100 um.

To validate the effect of HIFU on melanoma metastasis, we established a murine melanoma model and treated the mice with HIFU, following the protocol to simulate clinical operation. The median survival time and 95% confidence interval in the HIFU group was 26.00 days and 24.76~27.25 days, which was statistically higher than that of the control group, 19.00 days and 17.14~20.86 days, respectively (p<0.001, Figure 4A).

The target region of ablative HIFU in tumors became coagulative necrosis. There was increased leukocyte infiltration around the targeted region compared to that in the control group (Figure 4B). The lung metastasis incidence in HIFU-treated groups was 6.7% (1/15), which was much lower than that of the control group (20%, 3/15). The lung tissues in HIFU-treated mice were almost normal without visible foci, whereas it was grossly damaged with some tumor colonies in the control group (Figure 4C). The metastatic colonies were reduced by 40.7% in the lung tissue of HIFU-treated mice detected by histological analysis. These results confirm that HIFU is able to suppress distant metastasis of melanoma *in vivo*.

Then we investigated if the expression of miR-21 in tumor tissues was affected by HIFU. Some miRNAs that reported contribute to the pathogenesis or phenotypic metastatic behavior of melanoma were also examined [24–26]. MiR-21 was dramatically down-regulated after HIFU exposure (p<0.01) among the three down-regulated miR (miR-34a, −155, −222) (Figure 4D). These results confirmed that HIFU can alter expression of some miRNAs. Additionally, the expression of PTEN was increased and the activity of AKT pathway was suppressed (Figure 4E), confirming the *in vitro* results that HIFU suppresses the migration of B16-F10 cells via the miR-21/PTEN/AKT pathway.

Then we explored more mechanisms of HIFU's delayed effect on metastasis. Two weeks after HIFU treatment, the tumor tissues were resected from the mice in both groups to perform the further investigation. This time point was selected based on our previous observation that a two-week delay could maximize the recovery of host anti-tumor immunity [27]. In the tissues, we found that the protein level of E-cadherin (an epithelial marker) was elevated (p<0.05), and Vimentin (a mesenchymal marker) was reduced to some extent (p>0.05), and that VEGF and MMP9 were decreased (p<0.05) and the expression of MMP2 had no obvious change (data not shown). Thus, these findings suggested that the inhibitory effect of HIFU on lung metastasis of B16-F10 melanoma cells is partly mediated by suppressing EMT and reducing neovascularization and secretion of MMP9 (Figure 4F, 4G).

## DISCUSSION

The current mainstream therapies for melanoma include surgery, radiotherapy and chemotherapy, of which many are limited due to low response rates, severe toxicities and suppression of the host anticancer immunity [28]. HIFU is advantageous in these aspects. The beneficial observations in this study may be explained by thermal and non-thermal mechanisms of HIFU. The HIFU exposures used in this study caused that target tissues temperature elevated to more than 56°C for 1 second that generally leaded to instantaneous cell death via coagulation necrosis, which is the primary mechanism for tumor cell destruction in HIFU therapy. Subsequently, the tumor cell contents released form necrotic cells such as ATP and HSP60 as danger signals to stimulate subsequent anti-tumor host immunity, which are observed in animal and clinical studies [29–32]. From this viewpoint, the change of tumor microenvironment caused by HIFU exposure triggers a series of intricate molecular events that are critical for the therapeutic effects of HIFU.

While miRNAs are found to have an important role in radiotherapy [33], little is known about the involvement of miRNAs in HIFU treatment. Therefore, we speculated that miRNAs may play a role in this HIFU therapy and found four microRNAs that were inhibited by HIFU. Among these miRs, miR-21 was the one with the highest suppression by HIFU. MiR-21 is highly conserved in various mammalian species, implicating its important biological functions. Aberrant miR-21 expression is observed in melanoma tumor tissue samples and cell lines. Unrestricted proliferation, invasiveness and lack of apoptosis, which are all partly regulated by miR-21, have been seen in melanoma tumorigenesis. Thus, selective inhibition of miR-21 can be a potential therapy for melanoma [34–37]. MiR-21 is involved in the regulation of radiosensitivity. Down-regulation of miR-21 increased radiosensitivity in cancer cells.

As we all know, miR-21 has multiple target genes in melanoma. We hypothesized PTEN as one of miR-21 target genes, based on the following evidence: (1) PTEN-mRNA contains three putative miR21 seed motifs located in the 3′UTR region. (2) miR-21 affects pathways partially overlapping those regulated by PTEN, particularly those involved in cell migration [38, 39]. (3) Down-regulation of PTEN is associated with melanoma aggressiveness and worse prognosis in patients [40–44]. Many studies have demonstrated that microRNA targets are cell type-dependent. Therefore, we investigated and determined PTEN as a target gene of miR-21 in murine melanoma B16-F10 cells.

Various deletions in the PTEN gene have been identified in melanoma, the frequency of which is approximately 10% in primary melanoma and 40% in melanoma cell lines [45]. PTEN negatively regulates cell migration through direct dephosphorylation of the focal adhesion kinase and inhibition of MAP kinase and integrin-mediated cell spreading and migration [46–48]. Furthermore, PTEN can promote host immune response against cancer cells by repressing the expression of immunosuppressive cytokines IL-10, IL-6, and VEGF and programmed cell death 1 ligand (PD-L1) in a PI3K/AKT pathway-dependent manner [49, 50]. Recently, many studies reported that various drugs or chemicals up-regulate PTEN mRNA and protein expression to repress tumor formation and progression [51]. Furthermore, some studies have demonstrated that the PTEN/AKT pathway regulated the EMT procession by up-regulation of the transcription factors Snail and Twist [52–56]. In our study, we observed the putative relationship between the PTEN/PI3K pathway and the EMT procession after HIFU exposure. Our results support the role of PTEN and AKT in melanoma, which might be targeted by HIFU in treatment of melanoma. While we focused on PTEN in this report, miR-21 has other potential targets such as programmed cell death 4 (PDCD4) and P53. Further studies are warranted to investigate if other miR-21 target genes are involved in HIFU induced suppression of melanoma metastasis.

In summary, our findings provide evidence that HIFU has anti-metastatic effects on murine melanoma and one possible mechanism is through miR-21 down-regulation, increased PTEN expression and decreased AKT activity. Our study identifies a miR-21/PTEN/AKT pathway involved by HIFU for the inhibition of melanoma cell metastasis, which may be implicated in treatment of melanoma.

## MATERIALS AND METHODS

### Ethics

All animal work was performed in compliance with the guidelines established by the Chongqing Medical University Institutional Animal Care and Use Committee.

### Cell line and cell culture

Murine melanoma cell line B16-F10 was a kind gift from Dr. Yan Wang (College of Biomedical Engineering, Chongqing Medical University), which is derived from a spontaneous melanoma of C57BL/6J mice. All the cells were cultured in RPMI-1640 medium (Hyclone, USA) supplemented with 10% fetal bovine serum (Hyclone, USA), and 1% penicillin and streptomycin and routinely cultured at 37°C in a humidified atmosphere with 5% of CO_2_.

### Transfection of miR-21 mimic, inhibitor and plasmids

Murine miR-21 (GenePharma Co. Ltd, Shanghai) mimic, inhibitor or negative control was allowed to form transfection complexes with Lipofectamine™ 2000 in RPMI-1640 serum-free of serum and transfected into B16-F10 cells, according to the manufacturer's protocol.

The plasmids encoding an effective sequence to knock down the expression of murine PTEN (psiPTEN) and the scramble control siRNA sequence (psiCONTROL), were kindly presented by T.C. He (Medical Center, The University of Chicago). The transfection of plasmids was performed as above.

### Animals and tumor models

C57BL/6J female mice at 6–8 weeks of age were purchased from the Animal Center of Chongqing Medical University, housed with free access to food and water. B16-F10 cells (10^6^) were suspended in 100 μl of PBS and injected subcutaneously into the right back of the mice. Tumors were allowed to grow for about 7 days or their maximum diameter reached 7~10 mm and then the mice were allocated to two groups randomly, HIFU or sham-HIFU group (Control group), and then the mice underwent exposure.

### HIFU exposure system and HIFU treatment

The high focused ultrasound therapeutic apparatus set in this study is designed by Chongqing Haifu (HIFU) Medical Technology Co. Ltd (Chongqing, China) and is consisted of the main system, a power source, a transducer and a treatment pole. Ultrasound waves can be focused at a given point by the transducer and therefore magnified and delivered with precision to a small volume. Each mouse in the HIFU group received HIFU exposure and the exposure parameters were 1.6 MHz (frequency) and 4.5 W (acoustic power). Each focal point received a single exposure lasting 10 s [14, 15].

B16-F10 cells grown at log phase were resuspended with RPMI1640 medium and transferred into a 1.5 ml polyethylene centrifuge tube (2×l0^6^ cells/tube). The tube was immersed in a tank filled with degased water during the sonication. The HIFU energy was focused at the center of the tube. The HIFU group was sonicated for 1s, 2s or 3s at 142.7 W/cm^2^. The control group was with a fake HIFU sonication.

### Histology

Harvested samples were fixed in 4% formalin at 4°C, dehydrated in graded ethanol, embedded in paraffin, sectioned at 4 mm onto polylysine-coated slides, deparaffinized in xylene, rehydrated in graded ethanol, and stained with hematoxylin and eosin (H&E)[16].

mRNA/microRNA isolation, semi-quantitative RT-PCR and quantitative reverse transcription–polymerase chain reaction analysis (qRT-PCR).

Total RNA from each sample was reverse transcribed into cDNA and then RT-PCR was carried out as described (Takara RNA PCR kit) [4]. Mmu-miR-21primers (RT 5′-GTCGTATCCAGTGCAGGGTCCGAGGTATTCGCACTGGATACGACTCAACA-3′, forward 5′-TGGCGTAGCTTATCAGACTGA-3′, reverse 5′-GTGCAGGGTCCGAGGT-3′), mmu-miR-34a primers (RT 5′-GTCGTATCCAGTGCAGGGTCCGAGGTATTCGCACTGGATACGACACAACC-3′, forward 5′-GGTCTGGCAGTGTCTTAGCT-3′, reverse 5′-GTGCAGGGTCCGAGGT-3′), mmu-miR-155 primers (RT 5′-GTCGTATCCAGTGCAGGGTCCGAGGTATTCGCACTGGATACGACACCCCT-3′, forward 5′-GGCGTTAATGCTAATTGTGAT-3′, reverse 5′-GTGCAGGGTCCGAGGT-3′), mmu-miR-222 primers (RT 5′-GTCGTATCCAGTGCAGGGTCCGAGGTATTCGCACTGGATACGACAGGATC-3′, forward 5′-GCGCTCAGTAGCCAGTGTA-3′, reverse 5′-GTGCAGGGTCCGAGGT-3′), U6 primers (RT 5′-AAAATATGGAACGCTTCACGAATTTG-3′, forward 5′-CTCGCTTCGGCAGCACATATACT-3′, reverse 5′-ACGCTTCACGAATTTGCGTGTC-3′) were designed using the Primer5 program and synthesized by Invitrogen (Nanjing, China). The mRNA levels of PTEN were normalized to that of glyceraldehyde 3-phosphate dehydrogenase (GAPDH).

For quantitative analysis of miRNA expression, 2 ng of total RNA from each sample was used to generate cDNA with special stem-loop primer for each miRNA. Comparative real-time PCR was performed on the MyiQ Real-Time PCR Detection System (Bio-Rad) and the TaKaRa SYBR Green I premix was used. U6 was used as an endogenous control. These assays were performed following the manufacturer's instructions.

### Western blot

The protein was extracted from the residual tumor tissues for immunoblotting analysis. Briefly, the tissues were cut to pieces, homogenated using tissue homogenizer, and lysed in a buffer on ice. 200μg of proteins was separated in SDS-PAGE and then transferred to polyvinylidene difluoride (PVDF) membrane. After blocking at 37°C, the membranes were incubated with different primary antibodies at 4°C overnight. After washed with TBS supplemented with 0.1% Tween 20 (3×5 min), the membranes were incubated with secondary antibody for 1 h. Immunoreactive proteins were detected with enhanced chemiluminescence (Millipore Cor-poration, Billerica, MA, USA) using Bio-Rad Electrophoresis Documentation (Gel Doc 1000, Bio-Rad, USA) and Quantity One Version 4.5.0. The proteins were quantified and expressed as their ratio to β-actin.

### Immunohistochemical staining (IHC)

The paraffin-embedded tissue sections were deparaffinized and dehydrated. Then the sections were boiled for 15 min in 0.01 M citrate buffer. Endogenous peroxidase was blocked using 3% hydrogen peroxide for 30 min. Non-specific binding sites were blocked with 10% normal goat serum for 30 min at 37°C. Then the tissues were incubated at 4°C with primary antibodies overnight. Then the tissues were incubated with secondary antibody tagged with the peroxidase enzyme for 30 min at 37°C and were visualized with 2% 3, 3-diaminobenzidine tetrachloride (DAB) until the desired brown product was obtained. Finally the sections were counterstained with hematoxylin. The negative control group was carried out with the same steps as described above except replacing the primary antibody with phosphate buffer solution (PBS) [17]. All slides were observed under a OLYMPUS Light Microscope and representative photographs were taken.

### TUNEL assay

To detect apoptosis, tumor sections were assessed by terminal deoxynucleotidyl transferase-mediated dUTP nick end labeling (TUNEL) assay kit (*In Situ* Cell Death Detection Kit, POD, Roche Applied Science, Indianopolis, IN), according to manufacturer's instructions. Cells were considered positive when nucleus was stained brown. The number of apoptotic cells in a slide visualized under a high-power field (×400) was counted by two experienced pathological researchers.

### Luciferase reporter assay

Three of PTEN-3′UTR, which contains the three putative binding sites for miR-21, and PTEN-3′UTR-mut were synthesized by Invitrogen Incorporation (Nanjing, China). These segments were inserted into the pMIR-REPORT, miRNA Expression Reporter containing firefly luciferase, and pMIR-PTEN and pMIR-PTEN/mut were got, respectively. B16-F10 cells were cultured in 24-well plates, and 200ng of either pMIR-PTEN or pMIR-PTEN/mut was co-transfected with 200ng of pβ-gal, which contains β-galactosidase gene and was used for normalization of transfection differences, and 30nM of miR-21 mimic or control oligonucleotide. Transfection was performed using Lipofectamine 2000 (Invitrogen). Luciferase assays were performed at 48 hours after transfection by luciferase reporter assay system (Promega) and normalised by the β-galactosidase activity using the β-galactosidase Reporter Assay (Beyotime) according to the manufacturer's instructions. Three independent experiments were performed in triplicate.

### Trypan blue exclusion assay

Cell survival fraction was measured using the Trypan blue exclusion assay (Beyotime institute of Biotechnology). Cell suspension solution and Trypan Blue solution were mixed to stain non-viable cells. The viable and non-viable cells were counted under light microscope, and cell survival fraction (%) was calculated as [viable cells/(viable cells + non-viable cells)] × 100%.

### Cell viability assay by MTT [3-(4,5-dimethylthiazol-2-yl)-2,5-diphenyltrazolium bromide] assay

B16-F10 cells after HIFU sonication were seeded in 96-well plates (1000 cells/well) with RPMI-1640 containing 1% FBS for 48 h, and then the MTT reagent (Progema, Madison, WI, USA) was added (20μl/well). After incubation for 4 h at 37°C, 200μl dimethyl sulfoxide was added to dissolve formazan product for 10 min at room temperature. The absorbance was measured at 492 nm using a microplate reader. Each condition was done in triplicate, and the overall experiment was repeated thrice.

### Flow cytometry analysis (FCM)

The cells in each tube after HIFU sonication were cultured for 48h in flasks and then used for flow cytometry. Each flask was inoculated with 2×10^5^ cells. The apoptotic and necrotic cells were counted by flow cytometry (Beckman Coulter, Inc.). Vybrant apoptosis assay kit (Invitrogen detection technologies) was used. Cells were labeled with Annexin V and PI. Annexin V(−)/PI (−) was live cell, Annexin V(+)/PI (−) was apoptotic cell, and Annexin V(+)/PI(+) was dead cell.

### Wound healing assay

Cells after HIFU exposure were collected and seeded in 6-well plates. Each wound was created using a pipette tip at the center of the plate, then the cells were washed with serum-free medium, cultured with 1% FBS. Images were taken under a microscope immediately and at 48h after the incision. The wound healing rate was calculated as: (0h incision width - 48 h incision width)/0 h incision width × 100%.

### Transwell cell migration assay

The cells were seeded in the upper chamber contained 1% FBS. The bottom chamber was filled with RPMI-1640 containing 20% FBS as a chemo-attractant. After 48h, the cells were fixed with paraformaldehyde and stained with crystal violet. Finally the transmembrane cells were counted under a microscope at a power of 100. Five randomly selected fields in each well were counted. The experiments were repeated thrice. Representative photographs were shown.

### Statistical analysis

All experiments were performed three times, with at least three replicates per experiment. Data of only two groups were analyzed using Student's t-test. One-way ANOVA followed by the S-N-K test was used for the analyses of three or more groups. Statistical analysis was performed using the SPSS software version 17.0. Significant probability values were indicated as p<0.05*, p<0.01**, p<0.001***. A p value of less than 0.05 was considered significant.
